# Temporal-oscillatory entrainment: a multi-timescale framework for rhythmic coordination from neural to social frequencies

**DOI:** 10.3389/fnhum.2026.1806950

**Published:** 2026-06-18

**Authors:** Alex Gittelman

**Affiliations:** Boyer College of Music and Dance, Temple University, Philadelphia, PA, United States

**Keywords:** auditory-motor coupling, circadian rhythms, cross-frequency coupling (CFC), entrainment, music training, neural oscillations, neuroplasticity

## Abstract

**Background:**

The brain coordinates behavior across timescales spanning milliseconds to days through cross-frequency coupling (CFC)—the mechanism by which slow oscillations modulate the amplitude and timing of faster oscillations, creating a hierarchical temporal architecture. Existing frameworks have typically addressed oscillations within narrow frequency ranges, leaving cross-timescale coordination mechanisms underspecified.

**Approach:**

I propose a unified framework termed Temporal-Oscillatory Entrainment (TOE), which organizes entrainment phenomena (using the term in its chronobiological sense) across nine frequency ranges into three functional categories: neural (high gamma through delta), biological (behavioral through circadian), and social (weeks and longer). The framework identifies the auditory-motor system as a privileged pathway, given that CFC occurs in the auditory brainstem and rate-restricted coupling at approximately 4-5 Hz reflects an intrinsic auditory-motor rhythm.

**Evidence:**

Musical training studies demonstrate that rhythmic experience induces neuroplastic changes spanning subcortical to cortical levels, with transfer effects documented across multiple frequency bands—from millisecond-level discrimination to phrase-level processing. Beyond neural timescales, respiratory rhythms provide a well-characterized example of behavioral-frequency oscillations coupling to neural activity, while social rhythm therapy research demonstrates that stabilizing daily behavioral patterns affects circadian function and mood regulation. Musical ensemble coordination instantiates the same hierarchical nesting and phase-based coupling that characterizes neural CFC.

**Implications:**

The TOE framework generates testable predictions about bidirectional transfer effects between timescales, individual differences in entrainment capacity, and the distinct contributions of solo versus ensemble musical experience, with implications for understanding cognitive development, clinical intervention, and the neurobiological foundations of temporal coordination.

## Introduction

Rhythmic activity is a fundamental organizing principle of the nervous system (Buzsáki, 2006). Neural oscillations exist across species and recording methodologies, from the millisecond-scale spiking of individual neurons to the slow fluctuations of large-scale network activity (Buzsáki and Draguhn, 2004; Buzsáki and Vöröslakos, 2023). These oscillations are not epiphenomenal; rather, they reflect the coordinated activity of neural ensembles and are functionally implicated in sensory processing, attention, memory, and motor control (Buzsáki, 2006). As Buzsáki and Vöröslakos (2023) recently observed, “brain rhythms have come of age,” with neuronal oscillations now offering “access to neuronal operations, bringing microscopic and macroscopic mechanisms, experimental methods, and explanations to a common platform” (p. 922).

Existing neuroscience frameworks have typically addressed oscillations within narrow frequency ranges, leaving the mechanisms of this cross-timescale coordination underspecified. I propose a unified framework—Temporal-Oscillatory Entrainment (TOE)—that organizes the integration of entrainment phenomena across nine approximate frequency ranges into three functional categories. TOE specifies both the functional roles of individual frequencies and the mechanisms that enable their coordination. The framework’s title uses “entrainment” in the tradition of chronobiology (where the term encompasses any oscillator-to-zeitgeber synchronization) and the internal distinction within the framework between strict entrainment and oscillatory tracking operates within that broader usage.

The TOE framework synthesizes cross-frequency coupling research (e.g., Canolty and Knight, 2010; Hyafil et al., 2015b), auditory-motor neuroscience (e.g., Giraud and Poeppel, 2012; Assaneo and Poeppel, 2018), chronobiology (e.g., Schmidt et al., 2007), and social rhythm research (e.g., Frank et al., 2005) to propose testable mechanisms by which training at one timescale might affect processing at others.

A useful analogy to describe TOE emerges from musical ensemble performance. Existing research has characterized individual frequency bands in considerable detail—much as musicologists might analyze the range, timbre, and technical demands of individual instruments. Yet an ensemble is not a collection of soloists; music emerges from coordination across parts operating at different timescales, with performers synchronizing to one another while maintaining their distinct roles. The TOE framework adopts this organizing logic, specifying both the functional contributions of individual frequency bands and the entrainment mechanisms that enable their hierarchical coordination.

### Relationship to dynamic attending theory

The TOE framework builds upon Dynamic Attending Theory (DAT), which proposed that attention is inherently oscillatory and entrains to external temporal regularities (Jones, 1976; Jones and Boltz, 1989; Large and Jones, 1999). DAT transformed understanding of temporal cognition by replacing static models of attention with dynamic ones: attention fluctuates rhythmically, and when internal oscillations align with external rhythms, processing at expected timepoints is enhanced. Subsequent neural oscillation research has provided mechanistic grounding for DAT’s psychological claims, demonstrating that attentional entrainment reflects phase-alignment of neural oscillations to stimulus rhythms (Lakatos et al., 2008; Schroeder and Lakatos, 2009).

TOE extends this foundation in three directions. First, whereas DAT primarily addressed entrainment at single timescales, TOE proposes cross-frequency coupling as the binding mechanism that coordinates entrainment *across* timescales—from gamma-nested-in-theta at millisecond scales to circadian modulation of ultradian rhythms at hours-to-days scales. Second, TOE extends the hierarchy beyond neural oscillations to encompass biological frequencies (respiration, ultradian, circadian) and social frequencies (weekly, seasonal, developmental), proposing that the same coordinative logic operates across this extended range. Third, TOE identifies the auditory-motor system as a privileged pathway for engaging these mechanisms, given the anatomical instantiation of CFC in subcortical auditory structures. DAT asked how attention tracks time-varying events; TOE asks how the nervous system coordinates across the full temporal hierarchy, and how musical training might strengthen this coordination through its unique engagement of the auditory-motor pathway.

A terminological clarification is necessary before proceeding. The term “entrainment” appears in extant literature to describe two functionally distinct phenomena. Strict entrainment refers to phase-locking to isochronous external rhythms with simultaneous motor resonance—the phenomenon observed when a listener taps to a musical beat, when musicians synchronize to a shared pulse, or when a marching band generates coordinated movement (Savage et al., 2021). This form requires isochronous or near-isochronous rhythmic input and reliably engages the auditory-motor system. Oscillatory tracking, or intrinsic synchronicity (Meyer et al., 2019), refers to the endogenous adjustment of neural oscillatory phase to quasi-rhythmic input that lacks strict isochronous periodicity—the mechanism by which the brain processes speech, conversation, and other temporally structured but non-isochronous signals. Speech is not isochronous (Lehiste, 1977; Cummins, 2012), does not engage the motor system in the way music does, and is better described as eliciting oscillatory tracking than strict entrainment. Throughout this paper, “entrainment” is reserved for isochronous-beat phase-locking; “oscillatory tracking,” “synchronization,” or “coupling” are used for quasi-rhythmic neural coordination and for cross-frequency relationships more generally.

### Cross-frequency coupling: the mechanism of hierarchical coordination

Before detailing the frequency architecture of the TOE framework, it is necessary to establish the mechanism by which oscillations at different timescales interact. Cross-frequency coupling (CFC) refers to the statistical dependence between oscillatory activity at different frequencies (i.e., the means by which the brain coordinates processes operating at millisecond, second, and longer timescales into unified perception and action). Without such coupling, neural oscillations would be isolated phenomena, where each frequency band processes information in parallel but never integrates across timescales. CFC provides the binding mechanism that transforms a collection of oscillators into a hierarchically organized temporal architecture.

### The ensemble as mechanism, not metaphor

Earlier, I referenced a comparison between TOE and musical ensemble performance. Critically, this analogy is not merely pedagogical decoration; it reflects genuine structural correspondence between musical and neural coordination. An ensemble’s coordination is hierarchically nested: the conductor’s downbeat does not dictate every note but rather provides a temporal scaffold within which section leaders shape phrases and individual players execute rapid passages. The conductor operating at the slowest timescale (whole measures, phrase boundaries) does not micromanage the first violinist’s sixteenth-note runs. Instead, the conductor’s beat creates temporal windows, or moments of expected coordination, within which faster activity unfolds with local autonomy.

This is precisely the logic of phase-amplitude coupling (PAC), the most extensively studied form of CFC (Canolty and Knight, 2010). In PAC, the phase of a slow oscillation modulates the amplitude of a faster oscillation. The slow oscillation does not cause the fast activity; instead, it modulates when fast activity is most likely to occur, creating windows of heightened excitability within which faster processes can fire. For example, a theta oscillation (4–8 Hz) creates roughly 125–250 millisecond cycles; gamma activity (30–100 Hz) rises and falls within these cycles, clustering at particular phases. The theta wave is the conductor’s gesture; the gamma bursts are the rapid passages executed within that temporal frame.

Furthermore, coordination operates across spatial as well as temporal distance in a musical ensemble. The conductor synchronizes different instruments seated twenty meters (or larger, in the case of a marching band on a football field) apart—a distance too great for musicians to coordinate through direct auditory feedback at rapid timescales. The slow, visible conducting gesture propagates coordination across this spatial gap precisely because slow movements are perceptible across distance in ways that fast movements are not. Similarly, neural long-distance communication faces an analogous constraint. Fast oscillations attenuate over cortical distance; the precise timing of a gamma burst in the auditory cortex cannot directly synchronize with a gamma burst in the motor cortex. But slow oscillations (e.g., delta and theta rhythms) can maintain phase coherence across distributed networks, providing a temporal reference frame within which distant regions coordinate their faster local processing (Hyafil et al., 2015b). The conductor coordinates the ensemble by providing a shared temporal scaffold, just as slow neural oscillations coordinate distant brain regions through the same logic.

#### Functions of cross-frequency coupling

Hyafil et al. (2015b) identified three cognitive operations mechanistically implicated in CFC: multi-item representation, long-distance communication, and stimulus parsing. Each has a clear ensemble correlate, and recognizing these correspondences clarifies the functional logic of cross-frequency coordination.

*Multi-item representation*. Working memory requires maintaining multiple distinct items simultaneously—several digits of a phone number, several words in a sentence being parsed. The theta-gamma code proposed by Lisman and Jensen (2013) suggests that distinct items are represented by distinct gamma cycles nested within a single theta cycle. Each gamma burst encodes one item; the theta oscillation provides the temporal address system that keeps them sequentially organized rather than blurred together. In ensemble terms, this corresponds to a melodic line in which each note occupies a distinct position within the phrase’s arc. The phrase structure (theta) does not blur the notes into a continuous smear; it provides the hierarchical temporal framework within which each note (gamma) maintains its identity while contributing to the larger gestalt. A listener hears both the individual notes and the phrase they compose—just as working memory maintains both individual items and their sequential relationship.

*Long-distance communication*. As noted above, slow oscillations can maintain phase coherence across cortical distances that fast oscillations cannot. This property enables what Fries (2015) termed “communication through coherence”—the proposal that effective connectivity between brain regions depends on phase alignment of their oscillatory activity. When two regions oscillate in phase, their windows of excitability align, facilitating information transfer; when out of phase, communication is gated. Slow oscillations, by virtue of their ability to maintain long-distance coherence, set the temporal frame within which faster, spatially local processing occurs. The conductor again provides the visual reference: a gesture perceptible across the entire stage, enabling coordination that purely local, auditory-based synchronization could not achieve alone.

*Stimulus parsing*. The brain must segment continuous sensory input into discrete units for further processing—e.g., the speech stream into syllables and words, the musical stream into notes and phrases. Theta-gamma coupling provides a mechanism for this parsing: theta oscillations track the quasi-rhythmic structure of speech (syllable rate, approximately 4–8 Hz), while gamma-band activity provides windowed excitability within which rapid featural processing occurs (Giraud and Poeppel, 2012; Hyafil et al., 2015a). The slow oscillation marks boundaries; the fast oscillation processes content between boundaries. This arrangement is directly analogous to how listeners parse continuous melodies into discrete motives and phrases. The acoustic signal is continuous, but the perceived structure is segmented at phrase boundaries corresponding to delta and theta timescales. The listener does not consciously impose this structure; rather, the auditory system’s intrinsic oscillatory dynamics parse the input automatically, organizing continuous input into timescales appropriate for memory encoding.

#### Bidirectional influence across timescales

The discussion thus far has emphasized how slow oscillations modulate fast activity—the conductor shaping the ensemble’s temporal framework. But ensemble coordination is bidirectional, and so is cross-frequency coupling. A rhythm section’s precision at fast timescales (the drummer’s subdivisions, the bassist’s articulation) tightens the groove that enables the ensemble’s collective swing at slower timescales. A soloist’s rubato—local timing variations at the note level—can stretch or compress phrase-level timing, with the ensemble adjusting to follow. Information flows upward as well as downward through the temporal hierarchy.

Neural evidence supports this bidirectionality. While PAC canonically describes slow-phase modulation of fast amplitude, other coupling forms exist: amplitude-amplitude coupling, phase-phase coupling, and importantly, fast-to-slow influences in which precise fast activity sharpens slow oscillatory timing (Canolty and Knight, 2010). Training that enhances the precision of fast oscillatory activity, such as the millisecond-level motor timing demands of instrumental performance, could improve coupling with slower modulating rhythms. Conversely, interventions that strengthen or regularize slow oscillatory activity—such as entrainment to a musical beat at delta frequencies—could have cascading effects on faster timescales by providing a more stable temporal scaffold within which fast processes unfold.

This bidirectional influence is central to the TOE framework’s predictions about cross-timescale transfer. If slow and fast oscillations are reciprocally coupled, then training at any point in the hierarchy could propagate effects both upward and downward. Musical training is distinctive precisely because it engages multiple timescales simultaneously: the drummer maintaining a steady beat (delta), the melodic phrase shaping (theta), the precise onset timing distinguishing one articulation from another (gamma). This multi-timescale engagement may train not only each frequency band in isolation but the coupling mechanisms that bind them—a possibility examined in detail in subsequent sections on musical training and cross-frequency plasticity.

### Clinical significance of cross-frequency coupling

Evidence that CFC disruption characterizes multiple psychiatric and neurological disorders reinforces the proposal that coupling mechanisms are functionally significant rather than epiphenomenal. A systematic review by Yakubov et al. (2022) found that disrupted PAC patterns have been linked to autism spectrum disorder, schizophrenia, Parkinson’s disease, and other conditions involving temporal coordination deficits. The review noted that “stronger coupling tends to occur with higher neural computational needs” (p. 4), suggesting that CFC scales with processing demands and that its disruption impairs the integration required for complex cognition.

This clinical evidence has implications for intervention design. If CFC disruption contributes to symptomatology, then interventions that strengthen cross-frequency coordination might have therapeutic value. Musical training, with its documented effects on multiple frequency bands and its inherent demand for cross-timescale coordination, emerges as a candidate intervention—not because music is generically beneficial, but because it specifically engages the coupling mechanisms that appear disrupted in these conditions. This possibility is examined further in the discussion of clinical implications.

### Reading the TOE frequency architecture through coupling

The following sections detail each frequency range within the TOE framework—its neural generators, functional roles, and characteristic processing timescale. These sections can be read as a taxonomy, cataloging the oscillatory furniture of the brain. But, they are better read through the lens of cross-frequency coupling established here. At each frequency band, readers should attend to how that band couples to its neighbors: how gamma nests within theta, how theta coordinates with delta, how delta bridges to behavioral and circadian timescales. The individual frequency bands are instruments; CFC is the ensemble coordination that transforms them from parallel processes into an integrated temporal architecture.

The ensemble analogy, introduced here and recurring throughout, is not decorative. Musical ensemble performance instantiates—literally, not metaphorically—the hierarchical temporal coordination that CFC enables neurally. When musicians entrain to a shared beat, coordinate phrase-level timing, and synchronize millisecond-level onsets, they are exercising the same cross-frequency coordination mechanisms that organize perception, memory, and action. Understanding this correspondence illuminates both why musical training affects neural function across multiple timescales and why the TOE framework adopts ensemble coordination as its organizing logic.

This influence is anatomically specific. The frequency-following response (FFR) demonstrates that the auditory brainstem phase-locks to stimulus periodicity, with cross-frequency coupling occurring at subcortical levels (Coffey et al., 2016, 2021). Critically, the FFR is modulated by musical training (Musacchia et al., 2007; Wong et al., 2007), indicating that these subcortical mechanisms exhibit experience-dependent plasticity. While proprioceptive and visual modalities engage entrainment through cortical circuits, only auditory input directly accesses brainstem oscillatory mechanisms. This anatomical arrangement positions the auditory-motor system as a privileged pathway for training cross-frequency coupling—not exclusive, but uniquely direct.

### The frequency architecture of temporal-oscillatory entrainment

I use the TOE framework to organize entrainment phenomena across nine approximate frequency ranges further grouped into three functional categories: neural frequencies (high gamma through delta), biological frequencies (behavioral through circadian), and social frequencies. This taxonomy is not intended as a rigid classification but rather as an organizational model that highlights functional distinctions while acknowledging that the boundaries between frequency bands are gradual rather than discrete. Table 1 describes the full organization of the TOE framework.

**Table 1:** Organization of Temporal-Oscillatory Entrainment (TOE) Framework.

Category	Band	Frequency	Representative functions	Ensemble role
Neural	High Gamma	60–200 Hz	Onset detection, timbre	Articulatory detail
	Beta	13–30 Hz	Temporal prediction	Pulse-keeper
	Alpha	8–12 Hz	Sensory gating	Knowing when not to play
	Theta	4–8 Hz	Memory, phrase grouping	Phrase structure
	Delta	0.5–4 Hz	Prosody, inter-brain coupling	Conductor frequency
Biological	Behavioral	0.1–0.5 Hz	Respiration, turn-taking	Foundational ostinato
	Ultradian	~90–120 min	Arousal cycles, practice	Rehearsal-break structure
	Circadian	~24 h	Sleep–wake, restoration	Concert day
Social	Institutional/interpersonal	Weeks-years	Schedules, development	Career arc, ensemble membership

A note on frequency overlap between tiers is necessary, as the boundaries between categories are not sharp. Cardiac rhythm provides a useful example: resting heart rate (~1 Hz) falls squarely within the neural delta band (0.5–4 Hz), yet its primary generator is the sino-atrial node rather than neural oscillatory circuits. Like respiration, cardiac rhythm is categorized in the biological tier based on the timescale of its generating mechanism rather than its frequency alone. This overlap is a predicted consequence of the framework’s hierarchical nesting logic. The TOE framework proposes that slower biological oscillators interact with faster neural ones precisely at these frequency boundaries, creating zones of potential coupling rather than clean separations. The respiratory-neural coupling documented by Brændholt et al. (2023) and the cardiac-neural interactions documented in interoceptive processing research (Craig, 2009; Critchley and Harrison, 2013) both illustrate this boundary-zone coupling. Overlaps between tiers are therefore where the framework predicts the most interesting cross-timescale interactions, not where its taxonomy breaks down.

### Neural frequencies

The neural frequency category encompasses oscillations from approximately 0.5 Hz to 200 Hz, spanning delta through high gamma bands. These frequencies originate from well-characterized neural circuits examined through electrophysiological methods. A short survey of the neural architecture responsible for these frequencies, their associated functions, and theorized methods of coordination.

#### High gamma (60–200 Hz)

High gamma oscillations are generated through pyramidal-interneuron network interactions, arising from cycles of synchronous excitation followed by synchronous inhibition within local cortical circuits (Buzsáki, 2012). In the auditory domain, the primary neural generators are located in primary and nonprimary auditory cortex, including auditory association areas on the lateral superior temporal gyrus (Steinschneider et al., 2013). These responses emerge approximately 80–120 milliseconds after stimulus presentation, peaking around 100 milliseconds. Subcortical structures also contribute: the inferior colliculus generates frequency-following responses, while recent magnetoencephalography (MEG) work has demonstrated that the cortical contribution to the frequency-following response at approximately 100 Hz is stronger in the right hemisphere (Coffey et al., 2016; Bidelman, 2018). During cognitively demanding auditory tasks, the dorsal anterior cingulate cortex (dACC) shows enhanced high-gamma activity and increased functional connectivity with bilateral auditory cortices (Mulert et al., 2007).

Before characterizing gamma’s functional role in the TOE framework, a perceptual clarification is necessary. At auditory frequencies of 60 Hz and above, the perceptual regime shifts from rhythmic to spectral. Rather than a sequence of discrete events, the listener perceives tonal or noise-like qualities determined by periodicity and spectral composition (Albert and Grice, in press; see also Stockhausen, 1959, for an early musical treatment of this duality). This spectral regime is meaningfully distinct from the rhythmic regime that characterizes delta oscillatory coupling to beat and phrase structure. Gamma-band frequencies therefore do not participate in the TOE framework through perceptual rhythmic entrainment; that is, the sensorimotor coupling characteristic of slower oscillatory frequencies is not operative here.

What gamma contributes to the TOE framework is a different mechanism: phase-amplitude coupling (PAC). In PAC, the relevant quantity is not the gamma frequency itself but the envelope of gamma-band power—the fluctuating amplitude of fast activity that rises and falls within the cycles of a slower modulating oscillation. Critically, this amplitude envelope operates well within the rhythmic perceptual regime. The phase of a theta oscillation (4–8 Hz, cycling every 125–250 milliseconds) modulates when gamma amplitude is elevated, creating windows of heightened cortical excitability within which fast activity preferentially occurs. The spectral-to-rhythmic perceptual boundary does not constrain PAC because the coupling operates on the gamma amplitude envelope, not on gamma oscillations as a perceived rhythmic sequence. The conductor does not need to hear the first violin’s sixteenth notes as an entraining pulse—she gates when fast activity is most likely by providing a slow temporal reference frame within which it can unfold.

Functionally, high gamma oscillations are associated with fine temporal encoding, onset transient detection, and timbre processing. High gamma activity is particularly prominent during active listening and speech production, where gamma power modulates neuronal excitability in the superficial cortical layers that generate output signals toward higher processing stages. Computational modeling has demonstrated that gamma activity participates in a nested relationship with slower theta oscillations, creating what Buzsáki (2012) described as a hierarchical temporal code in which fast assembly activity is organized by slower oscillatory sequences. Hyafil et al. (2015a) demonstrated through neural microcircuit modeling that theta-gamma coupling provides a viable mechanism for hierarchically organizing rapid featural processing within slower oscillatory cycles—a coupling architecture whose functional application to speech encoding is taken up separately from the framework’s structural claims.

It is also worth noting a limitation of the discretization reading of this work. Interpreting gamma as parsing continuous speech into atomic phoneme symbols encounters difficulties in the phonetics literature: coarticulation, reduction, and resyllabification mean the acoustic signal does not contain clean phoneme-sized units awaiting segmentation (Fowler, 1980; Cangemi and Niebuhr, 2018). More fundamentally, Christiansen and Chater's (2016) now-or-never bottleneck argues that stringing atomic symbols into sequences before accessing meaning is computationally implausible given processing-time constraints. Importantly, the TOE framework does not require this discretization claim as the relevant point is architectural: gamma-band activity provides rapidly fluctuating excitability windows nested within slower oscillatory cycles, and this nesting enables a hierarchical code for temporal structure across timescales. Whether the outputs of that fast processing are best described as phoneme-like symbols or as graded featural representations is a question separate from the coupling architecture that the TOE framework describes.

Within the musical ensemble analogy, high gamma functions as fine articulatory detail—the attack transients and timbral characteristics that distinguish instruments within a complex texture, or the rapid acoustic events that mark note onsets. These fast events are not themselves the subject of rhythmic entrainment, but their precise timing is gated by the slower oscillatory structure within which they are embedded.

#### Beta (13–30 Hz)

Beta oscillations arise from two principal sources: the sensorimotor cortex and the basal ganglia (Spitzer and Haegens, 2017). In the sensorimotor cortex, beta oscillations are generated by corticospinal-projecting pyramidal neurons in layer 5 (Roopun et al., 2006; Yamawaki et al., 2008). The basal ganglia, including the putamen, globus pallidus, and subthalamic nucleus, constitute the second major source, with beta activity propagating through the cortico-basal ganglia-thalamic loop (Nevado Holgado et al., 2010; McCarthy et al., 2011). Additional generators include the supplementary motor areas, premotor cortex, and cerebellum for timing-related functions.

A perceptual clarification applies here as well. The beta band (13–30 Hz) occupies an auditory transition zone between the rhythmic and spectral perceptual regimes. At beta frequencies, isochronous sequences are too fast for listeners to perceive them as a rhythmic pulse—the perceptual lower boundary for pitch emergence begins to encroach at the upper end of this range. Beta therefore does not participate in the TOE framework through perceptual rhythmic entrainment to external beta-frequency stimuli; instead, beta oscillations support internally generated temporal predictions rather than phase-locking to an external rhythmic source.

Extant literature provides the mechanistic basis for this endogenous rhythmic function. Recordings from the putamen in macaques during rhythmic synchronization tasks reveal that beta-band local field potentials show “interval tuning,” with preferential responses to intervals around 800 milliseconds (Bartolo et al., 2014; Merchant et al., 2013). Crucially, this interval tuning appears specifically during internally-driven continuation timing rather than sensory-guided synchronization, thereby establishing that beta tracks an endogenous temporal prediction rather than an externally imposed rhythm. Beta oscillations are prominent during stable postures and decrease during active movement, consistent with the proposal that they reflect a state of “neuronal activity equilibrium” or predictive readiness (Engel and Fries, 2010; Spitzer and Haegens, 2017). The clinical observation that excessive beta in Parkinson’s disease correlates with rigidity and slowed movement supports the interpretation that beta maintains a temporal status quo that must be released for movement to proceed—a function analogous to holding a predicted beat interval until the next event confirms or updates the prediction.

Direct evidence that beta tracks auditory beat structure comes from Fujioka et al. (2015), who found that beta oscillations represent auditory beat and its metrical hierarchy in both perception and imagery, with musicians showing enhanced responses. This finding establishes beta’s role as both a motor timing mechanism and a carrier of hierarchical temporal scaffolding across the metrical hierarchy. Doelling and Poeppel (2015) further showed that cortical entrainment to music at delta and theta frequencies is modulated by beta-band expertise effects, suggesting that beta-mediated beat prediction is coupled to (and may scaffold) the slower oscillatory tracking of phrase structure.

In a musical ensemble, beta oscillations function as the pulse-keeper in a specific and technically grounded sense: as an endogenous predictive mechanism that maintains a stable interval representation against which beat-level and phrase-level structure can be organized. The beta system holds the beat internally between external events, enabling musicians to stay synchronized during rests, tempo changes, and moments of structural ambiguity where the external signal temporarily underspecifies the meter.

#### Alpha (8–12 Hz)

Alpha oscillations arise from thalamic and cortical sources, and their relative contributions depend on one’s behavioral state. While Andersen and Andersson (1968) established the thalamus as the primary pacemaker of alpha rhythm, subsequent *in vitro* and *in vivo* studies revealed that pyramidal neurons in the deep layers of sensory cortices can oscillate independently in the alpha frequency range (Lopes da Silva, 1991; Silva et al., 1991). Laminar recordings from macaque striate cortex, combined with Granger causality analysis, implicate a thalamocortical interaction loop involving layer 6 → lateral geniculate nucleus (LGN) → layer 4C → layer 6 as the primary alpha-generating circuit (Bollimunta et al., 2011). At the single-cell level, thalamocortical relay neurons exhibit periodic bursting in the alpha range during hyperpolarization of the membrane potential, a state associated with decreased sensory transmission most notably during sleep (Steriade et al., 1990). This cellular mechanism has led to the hypothesis that alpha oscillations implement gating at the thalamic level (Lopes da Silva, 1991).

Functionally, alpha oscillations implement sensory gating through rhythmic inhibition of neuronal spiking. Studies in monkey sensorimotor cortex have demonstrated that alpha oscillations produce pulsatile inhibition, with neuronal firing suppressed at specific phases of the alpha cycle (Haegens et al., 2011). In humans, increased alpha power over regions representing to-be-ignored information reflects active suppression of distractors during selective attention (Foxe and Snyder, 2011). This suppressive function has been demonstrated across multiple attention paradigms. For example, alpha power is greater over visual cortex when preparing to attend to auditory rather than visual stimuli (Foxe et al., 1998), and alpha lateralization over posterior parietal and occipital regions tracks the direction of spatial attention, with higher alpha power contralateral to the to-be-ignored hemifield (Worden et al., 2000). Klimesch (2012) proposed that alpha synchronization is associated with active inhibition of task-irrelevant information, while alpha desynchronization reflects release from inhibition during active processing. The attention-related modulation of alpha is predominantly under control of a cortico-cortical network involving frontal and parietal regions, with a secondary influence from thalamocortical connections—contrasting with arousal-related alpha, which is more strongly thalamus-dominated (Foxe and Snyder, 2011). In a musical ensemble, this manifests when a musician must know when (and when not) to play; alpha oscillations provide this function neurally, gating task-irrelevant streams so that attention remains focused on contextually appropriate input.

#### Theta (4–8 Hz)

Theta oscillations are generated primarily in the hippocampus, which researchers have recognized as the main structure involved in the 4–8 Hz theta rhythm (Buzsáki, 2002). The hippocampal theta rhythm emerges from interactions between two types of input: extrinsic pacemaking signals from the medial septum-diagonal band of Broca (MS-DbB), and intrinsic oscillatory activity generated within the hippocampus and entorhinal cortex (EC). The MS-DbB provides the theta pacemaker through cholinergic and GABAergic projections, while EC layers II and III provide rhythmic input via the perforant and temporoammonic pathways, respectively (Colgin, 2013). Within the hippocampus, CA3 activity reaches CA1 stratum radiatum through the Schaffer collaterals, contributing to the characteristic amplitude and phase variation of theta across hippocampal layers. Additional theta generators have been identified in the prefrontal cortex, where frontal midline theta emerges during working memory and executive function tasks (Raghavachari et al., 2006; Mitchell et al., 2008), and in the mediodorsal thalamus.

Theta oscillations are strongly associated with memory function, with enhanced theta during successful episodic memory encoding and retrieval (Herweg et al., 2020). As discussed in the preceding section, theta provides the temporal scaffolding within which gamma-mediated content is organized—a mechanism that enables ordered sequences of items to be maintained in working memory. Beyond this coupling function, theta oscillations serve as a timing mechanism to temporally organize movement sequences and planned trajectories for spatial navigation (Colgin, 2013). Theta synchronization between the hippocampus and neocortical regions, including prefrontal cortex and sensory areas, mediates the retrieval of multiple types of memory (Herweg et al., 2020). Theta thus serves a function analogous to musical phrase grouping—tracking where musical sentences begin and end, chunking the continuous stream of events into semantically meaningful units that can be held in working memory and integrated with what came before.

#### Delta (0.5–4 Hz)

Delta oscillations arise in the bilateral auditory cortex, frontal cortex, and speech-motor integration areas including superior and middle temporal regions (Ghitza, 2021). Bourguignon et al. (2013) used MEG studies to show that delta-band activity in the auditory cortex tracks speech prosody through oscillatory phase alignment (cf. Meyer et al., 2019). The insula has also been implicated in delta-range activity related to prosodic and interoceptive integration, given its role in coordinating internal bodily rhythms with external temporal structure. Delta oscillations can be elicited by prosodic chunk rates that fall within the delta frequency band (0.5–2 Hz). These oscillations have strong periodicities observed in the superior and middle temporal areas and speech-motor integration areas when chunk rates are within this range, and diminished or absent periodicities when chunk rates fall outside delta (Ghitza, 2021).

Delta-band activity is modulated by linguistic structure beyond acoustic prosody. Using mutual information analysis of EEG responses to naturalistic speech, Martin et al. (2020) found that delta-band activity was highest for sentences at phrasal timescales (0.8–1.1 Hz) and lexical timescales (1.9–2.8 Hz), suggesting that “linguistic content (i.e., structure and meaning) organizes neural oscillations beyond the timescale and rhythmicity of the stimulus” (p. 9467). This conclusion indicates that delta oscillations reflect endogenous, top-down linguistic processing. Meyer et al. (2017) demonstrated that delta-band oscillatory phase can be modulated by top-down linguistic grouping biases independently of acoustic prosodic cues, further supporting this interpretation. When listeners formed phrases that contradicted speech prosody due to syntactic ambiguity, their delta-band phase reflected their chosen linguistic grouping rather than the acoustic structure, demonstrating that “an internal linguistic bias for grouping words into phrases can modulate the interpretational impact of speech prosody via delta-band oscillatory phase” (p. 4293).

Delta frequencies appear to serve as a critical bridge between neural and behavioral timescales. During ensemble musical performance, inter-brain connections primarily operate at delta and theta frequencies, while intra-brain connections utilize faster beta frequencies (Müller et al., 2012; Müller and Lindenberger, 2013). The ensemble analogy here shifts from metaphor to mechanism. Müller and colleagues’ findings demonstrate that when musicians perform together, their coordination literally operates through delta-theta inter-brain coupling—slower oscillations synchronize activity between individuals while faster oscillations coordinate processing within individuals. Delta thus functions as a conductor or “hub frequency”: not dictating what each player does, but providing the temporal reference structure that allows independent parts to cohere into unified performance.

The proposal that delta serves as a “hub frequency” connecting neural processing to behavioral and social timescales is further supported by evidence that delta-band oscillatory responses to speech structure are present from birth (Ortiz-Barajas et al., 2023). This early neural sensitivity is consistent with the evidence reviewed above: delta tracks prosodic chunk boundaries (Ghitza, 2021), with Inbar et al. (2025) providing cross-linguistic converging evidence that intonation units form low-frequency rhythms within the delta range—suggesting this neural-prosodic alignment may reflect a universal property of speech timing. Delta also reflects hierarchical linguistic structure determined by meaning and syntax rather than acoustic rhythmicity alone (Martin et al., 2020), and its phase reflects top-down linguistic grouping independently of acoustic prosody (Meyer et al., 2017). Together, these findings ground delta’s role in hierarchical linguistic processing without requiring claims about cross-linguistic rhythmic discrimination, which remains contested in the phonological literature (e.g., Arvaniti, 2009; Nolan and Jeon, 2014).

### Biological frequencies

The ensemble analogy extends beyond neural frequencies. Just as musicians coordinate not only within a single performance but across rehearsals, seasons, and careers, temporal coupling phenomena operate at behavioral and social timescales that reach well beyond the sub-second dynamics of neural oscillations. The following sections examine how the same coordinative logic (e.g., hierarchical nesting, phase-based coupling, mutual synchronization) manifests in biological rhythms. The biological frequency category encompasses oscillations slower than delta, ranging from behavioral frequencies (0.1–0.5 Hz) through ultradian rhythms to circadian cycles. These frequencies are less commonly studied in cognitive neuroscience; however, they represent fundamental organizing principles of behavior and physiology.

Evidence for cross-timescale transfer at biological frequencies is currently less extensive than at neural frequencies, reflecting both methodological challenges in studying slower rhythms and the relative novelty of integrating chronobiology with oscillatory neuroscience. The following sections synthesize available evidence while identifying specific gaps the TOE framework predicts should be addressable.

### Behavioral frequencies (0.1–0.5 Hz)

Behavioral frequencies encompass the timescale of phrasing, turn-taking in conversation, and movement sequences—the temporal grain at which organisms interact with their environment as integrated wholes rather than as collections of neural processes. This frequency range marks a critical transition in the TOE framework: while neural frequencies arise from well-characterized circuits within the brain, behavioral frequencies emerge from the interaction of neural systems with bodily processes that extend beyond the nervous system proper.

The neural architecture supporting behavioral-frequency oscillations involves distributed networks operating across cortical and subcortical structures. The default mode network (DMN), including medial prefrontal cortex, posterior cingulate cortex, and angular gyrus, shows intrinsic fluctuations in the 0.1 Hz range during rest (Raichle et al., 2001). The anterior cingulate cortex (ACC) integrates information across cognitive, affective, and motor domains at timescales consistent with behavioral frequencies, while the orbitofrontal cortex (OFC) tracks value and outcome information that unfolds over seconds to tens of seconds (Padoa-Schioppa and Conen, 2017). The insula serves as a critical hub at this timescale, integrating interoceptive signals, including respiratory, cardiac, and visceral state, with exteroceptive processing to generate a continuously updated representation of bodily selfhood (Craig, 2009; Critchley and Harrison, 2013).

Respiration provides the most extensively documented example of behavioral-frequency oscillations coupling to neural activity, but a clarification about the TOE framework’s taxonomy is warranted here. Respiration is placed in TOE’s “biological” tier because its primary generator (the pre-Bötzinger complex and associated brainstem respiratory circuits) operates at approximately 0.2–0.5 Hz, below the conventional lower boundary of delta (~0.5 Hz). This categorization reflects the timescale of the generating mechanism, not a claim that respiratory-cortical coupling operates outside neural oscillatory frameworks; rather, that coupling is thoroughly mediated by neural oscillatory mechanisms. Varga and Heck (2017) proposed that respiration serves as a “global oscillatory scaffold” that organizes faster neural rhythms, with gamma and theta power modulated by respiratory phase. Brændholt et al. (2023) confirmed using high-density EEG that respiratory phase systematically modulates the amplitude of delta, theta, and alpha oscillations across widespread cortical regions in humans. The TOE framework incorporates these findings: what this framework adds is the observation that this neural-oscillatory coupling mechanism extends the hierarchical nesting logic downward across the neural-biological boundary.

Zelano et al. (2016) demonstrated that nasal respiration phase-couples to oscillatory activity in human piriform cortex, amygdala, and hippocampus, with cognitive consequences: participants showed enhanced fear discrimination and memory retrieval specifically during inhalation, when limbic oscillatory power was maximal. Critically, these effects were specific to nasal breathing and absent during oral respiration, implicating the olfactory system as a conduit for respiratory-brain coupling. However, subsequent work by Perl et al. (2019) revealed that non-olfactory cognitive processes (e.g., visuospatial attention and reaction time) also phase-lock to respiration even during mouth breathing. These findings suggest multiple pathways through which respiratory rhythm modulates cognition. As Boyadzhieva and Kayhan (2021) summarized in their comprehensive review, “rhythmic breathing exerts a backward influence on the brain,” with respiration-coupled oscillations observed across hippocampus, prefrontal cortex, amygdala, and sensorimotor regions.

This cross-frequency coupling positions respiration as a bridge between neural and biological timescales. The respiratory frequency range (0.2–0.5 Hz) shares the lower boundary of delta oscillations, creating a zone of potential interaction. Just as theta oscillations provide a temporal scaffold for nested gamma cycles in the hippocampus, respiration may function as a slower oscillation within which delta and theta activity is hierarchically organized. What Tort et al. (2018) characterize as ‘breathing entrains the brain’, understood within the TOE framework as respiratory-neural phase coupling rather than isochronous beat entrainment, thus extends the principle of cross-frequency coupling across the boundary between subcortical brainstem oscillators and forebrain neural oscillations. This framing suggests that the hierarchical nesting observed within the brain continues outward to encompass bodily rhythms.

In ensemble terms, respiration operates as a foundational ostinato—a repeating pattern that runs beneath conscious awareness but consistently shapes the temporal structure available to other, more flexible processes. Unlike the higher-frequency neural oscillations that demand continuous coordination, the respiratory rhythm provides a stable, semi-autonomous pulse to which other processes can synchronize without conscious effort. Respiration, like a virtuoso musician, is not rigidly mechanical: respiration adjusts to speech, emotion, physical exertion, and voluntary control, demonstrating the bidirectional coupling between behavioral and neural timescales.

The relationship between respiration and speech prosody is well-established: respiratory cycles segment speech into intonational and phrasal units, with the neural mechanisms of this coupling documented in the literature cited above. Whether a comparable coupling operates during musical performance is less well-established and warrants explicit acknowledgment. The evidence that performers regularize breathing around phrase boundaries is well-documented at the level of pedagogical practice and performance observation, but controlled empirical study of respiratory-neural coupling specifically during music making remains limited relative to the speech literature. The TOE framework predicts that such coupling should be detectable, as the hierarchical nesting logic that applies to speech phrasing should extend to musical phrasing, given the comparable timescales involved, but this prediction awaits direct empirical confirmation. The framework treats this as a productive open question rather than an established result.

Beyond respiration, other behavioral-frequency phenomena warrant investigation within the TOE framework. Conversational turn-taking operates at approximately 0.2–0.5 Hz, with speakers exchanging turns every 2–5 s on average (Levinson and Torreira, 2015). This temporal structure falls within the range of respiratory cycling, suggesting that the rhythm of conversation may be constrained by the same biological oscillation that modulates individual cognition. Similarly, spontaneous movement sequences, postural adjustments, and eye movement patterns show temporal organization in the behavioral frequency range (Gilden et al., 1995), hinting at a common oscillatory infrastructure underlying diverse forms of behavior.

Just as delta oscillations provide a temporal scaffold for faster neural frequencies, behavioral-frequency rhythms like respiration may themselves be modulated by slower ultradian cycles. This nesting principle (the signature organizational logic of the TOE framework) suggests that the hierarchy extends beyond the boundary between neural and biological timescales into the domain of ultradian and circadian rhythms.

### Ultradian rhythms (~0.0001–0.0003 Hz; 90–120 min)

Ultradian rhythms occupy the timescale between behavioral frequencies and circadian cycles, governing fluctuations in arousal, attention, and cognitive performance across periods of approximately 90–120 min. Kleitman (1963) first proposed the Basic Rest-Activity Cycle (BRAC) based on observations that the ~90-min periodicity of REM sleep continues into waking hours, manifesting as cyclic variations in alertness, perceptual sensitivity, and cognitive efficiency. While this proposal has subsequently received substantial empirical support, the mechanisms remain incompletely understood.

The neural substrates of ultradian rhythms involve hypothalamic circuits, brainstem arousal systems, and neuromodulatory pathways. The locus coeruleus noradrenergic system and dorsal raphe serotonergic system show phasic activity patterns that modulate cortical arousal states (Aston-Jones et al., 2001). The hypothalamus coordinates ultradian fluctuations through orexin/hypocretin neurons that regulate sleep–wake transitions and interact with circadian signals from the suprachiasmatic nucleus (Saper et al., 2005). Endocrine systems exhibit prominent ultradian pulsatility: growth hormone, cortisol, and luteinizing hormone are secreted in ~90–120 min pulses rather than continuously, with this pulsatile pattern essential for normal physiological function (Veldhuis et al., 1989).

Evidence for waking ultradian rhythms in cognition comes from multiple paradigms. Chapotot et al. (1998) demonstrated ~90-min fluctuations in EEG spectral power during sustained wakefulness, with these oscillations becoming more prominent under sleep deprivation when homeostatic sleep pressure unmasks the underlying ultradian structure. Studies of sustained attention reveal performance decrements following approximately 90-min periods of continuous work, with brief rest intervals restoring performance (Folkard and Tucker, 2003). Intriguingly, research on expert performance converges on similar timescales: Ericsson et al. (1993), in their influential study of deliberate practice, found that elite performers across domains typically structure practice into sessions of approximately 90 min, rarely exceeding this duration without rest. This convergence between optimal practice structure and the BRAC period suggests that effective skill acquisition likely requires alignment with ultradian arousal cycles.

The nesting relationship between ultradian and adjacent timescales follows the hierarchical logic of the TOE framework. Just as respiratory rhythms (behavioral frequencies) provide a scaffold for faster neural oscillations, ultradian rhythms may modulate the overall envelope of arousal within which behavioral-frequency processes operate. A musician’s respiratory synchronization with phrase structure, for example, occurs against a backdrop of ultradian fluctuations in alertness and attentional capacity. Similarly, ultradian rhythms are themselves nested within the circadian cycle: BRAC periodicity is modulated by time of day, with some evidence suggesting stronger ultradian oscillations during circadian troughs in alertness (Monk, 2005).

In musical ensemble terms, ultradian rhythms function as the rehearsal-break structure of cognitive life—the alternation between focused engagement and recovery that enables sustained performance across a rehearsal and the day as a whole. Just as effective ensemble rehearsal requires periodic breaks to consolidate learning and restore attention, cognitive function depends on ultradian cycling between high and low arousal states. Musical practice traditions, often developed through centuries of accumulated pedagogical wisdom, may implicitly honor these biological constraints.

### Circadian rhythms (~1.16 × 10^−5^ Hz; ~24 h)

Circadian rhythms govern the temporal organization of physiology and behavior across the 24-h day. Unlike neural oscillations that emerge primarily from synaptic interactions, circadian rhythms arise from transcription-translation feedback loops operating within individual cells, coordinated by a central pacemaker in the suprachiasmatic nucleus (SCN) of the hypothalamus.

The molecular clock mechanism involves interlocking feedback loops of clock genes. The transcription factors CLOCK and BMAL1 drive expression of Period (Per1, Per2, Per3) and Cryptochrome (Cry1, Cry2) genes; the protein products of these genes then accumulate, dimerize, and inhibit their own transcription by repressing CLOCK-BMAL1 activity (Hastings et al., 2018). This cycle takes approximately 24 h to complete. The SCN contains roughly 20,000 neurons, each an autonomous oscillator, but intercellular coupling synchronizes them into a coherent tissue-level rhythm that is more robust than any individual cell (Welsh et al., 2010). The SCN communicates timing information to the rest of the brain and body through multiple pathways: neural projections to hypothalamic nuclei controlling sleep, feeding, and hormone release; humoral signals including the nocturnal release of melatonin from the pineal gland; and autonomic outputs to peripheral organs (Dibner et al., 2010). Peripheral clocks in liver, heart, muscle, and other tissues maintain their own circadian oscillations, synchronized by SCN-derived cues but also responsive to local signals such as feeding times. This organization creates a hierarchical temporal architecture in which the SCN serves as conductor of distributed oscillators throughout the body.

Circadian rhythms profoundly influence cognitive function (i.e., TOE’s neural levels). A recent comprehensive review noted that “circadian rhythms act directly on human cognition and indirectly through their fundamental influence on sleep/wake cycles” (Cajochen and Schmidt, 2025). Long-term memory formation shows circadian modulation, with the hippocampus exhibiting daily oscillations in synaptic plasticity-related signaling cascades. Eckel-Mahan et al. (2008) demonstrated that MAPK (mitogen-activated protein kinase) pathway activity in the hippocampus oscillates with circadian periodicity, and that disrupting this oscillation impairs memory consolidation. Ruby et al. (2008) found that surgically ablating the SCN in aged hamsters (thereby eliminating circadian rhythms) actually rescued memory deficits, suggesting that a degraded or “noisy” circadian signal may be more detrimental to cognition than no signal at all.

Circadian rhythms also modulate attention, executive function, and emotional regulation across the day. These cognitive fluctuations interact with ultradian cycles: the ~90-min BRAC operates against a circadian backdrop of varying arousal, with ultradian periodicity more pronounced during circadian troughs in alertness (Monk, 2005). Critically, circadian rhythms are essential for health and physiological restoration—the nocturnal period involves tissue repair, immune function consolidation, and metabolic recalibration that cannot occur effectively when circadian organization is disrupted. Shift work, jet lag, and social jet lag (misalignment between social schedules and biological time) are associated with increased risk of metabolic syndrome, cardiovascular disease, and mood disorders (Baron and Reid, 2014).

Research on rhythmic auditory stimulation has documented effects on circadian markers. Kumar et al. (1999) found that 4 weeks of morning music therapy sessions in patients with Alzheimer’s disease resulted in marked increases in serum melatonin levels, suggesting that structured rhythmic input can strengthen circadian amplitude. While these studies primarily involve passive listening rather than active musical training, they raise the possibility that entrainment at auditory timescales (delta, theta) propagates upward through the TOE hierarchy to influence circadian organization. The mechanism of this cross-timescale transfer remains speculative but likely involves the reticular activating system, which both modulates arousal in response to auditory input and receives circadian modulation from the SCN.

The nesting relationship between circadian and adjacent timescales completes the biological tier of the TOE framework. Ultradian rhythms (~90–120 min) operate as subdivisions within the circadian day, their expression modulated by circadian phase. Behavioral-frequency oscillations (respiration, movement) and neural oscillations are, in turn, nested within ultradian arousal cycles. Sleep provides the clearest example of this hierarchical organization: the circadian system gates sleep timing, within which ultradian NREM-REM cycles unfold, within which neural oscillations—slow oscillations, sleep spindles, hippocampal sharp-wave ripples—coordinate memory consolidation at millisecond-to-second timescales (Diekelmann and Born, 2010). Musical engagement during waking hours may represent an analogous hierarchical coordination, with practice sessions (ultradian) embedded within daily schedules (circadian), and neural entrainment to musical structure embedded within practice sessions. In ensemble terms, the circadian rhythm structures the concert day—the arc from preparation through performance to recovery. A professional musician’s day involves circadian-appropriate timing of practice (often morning, when cortisol supports alertness and motor learning), performance (evening, when fine motor coordination and emotional expression peak for many individuals), and rest (night, when memory consolidation occurs). The circadian system thus provides the temporal scaffold within which musical activities (e.g., rehearsing, performing, listening, learning) are embedded.

### Social frequencies (weeks to years)

Social frequencies occupy the slowest tier of the TOE framework, encompassing rhythmic structures that emerge from the coordination of behavior across individuals and institutions: weekly schedules, monthly cycles, seasonal patterns, annual routines, and the developmental trajectories that unfold across years. These rhythms differ fundamentally from neural and biological oscillations in their generative mechanisms. They arise from social coordination rather than cellular or circuit-level processes, yet these rhythms exhibit the same organizational logic of hierarchical nesting and coordination that characterizes faster timescales.

A terminological clarification is necessary as the framework extends to social timescales. At neural and biological levels, “oscillation” carries a precise mechanistic meaning: recurring activity with defined phase, amplitude, and frequency, generated by identifiable cellular or circuit-level processes and measurable through electrophysiological or physiological methods. Cross-frequency coupling at these levels involves technically specified relationships between phase and amplitude that are empirically tractable. At social timescales, the term is used differently. Weekly rehearsal schedules, seasonal concert cycles, and career trajectories are not oscillations in this technical sense as they lack the mechanistic precision of neural oscillations and cannot be described in terms of phase-amplitude coupling in any direct way. The TOE framework uses “rhythmic regularity” as the appropriate descriptor at social timescales: recurring patterns of behavior and coordination that exhibit periodicity and coordinative function without the mechanistic substrate that defines oscillations at faster timescales. The framework’s claim at social timescales is architectural rather than mechanistic—that these rhythmic regularities exhibit the same organizational logic as neural oscillations (hierarchical nesting, bidirectional influence across timescales, coordinative function between individuals and institutions) rather than the same cellular or molecular mechanisms. This distinction between oscillatory mechanisms and rhythmic regularities maps onto the PAC caveat already articulated in the Theoretical Contributions: the framework proposes that coordinative principles extend across timescales, not that identical mechanisms operate at every level of the hierarchy.

The neural substrates supporting social temporal coordination involve distributed networks rather than localized pacemakers. The medial prefrontal cortex (mPFC) and temporoparietal junction (TPJ) form a core network implicated in mentalizing, social prediction, and processing others’ intentions—functions essential for coordinating behavior across individuals and anticipating socially structured time (Frith and Frith, 2006). Premotor and inferior parietal regions implicated in action observation and imitation support the behavioral synchronization that enables temporally coordinated interaction (Iacoboni, 2009). Feldman (2007) proposed that the capacity for temporally coordinated social interaction is grounded in physiological oscillator systems, including circadian and cardiac pacemakers, along with attachment-related neuroendocrine systems such as oxytocin. This “biobehavioral synchrony” model suggests that social rhythms emerge from the coordination of biological rhythms between interacting individuals, with the oxytocin system facilitating the coupling.

Parent-infant synchrony provides a developmental foundation for social-frequency coordination. From the earliest months of life, caregivers and infants engage in temporally coordinated exchanges of gaze, vocalization, and affect—proto-conversations that exhibit turn-taking structure at behavioral frequencies (~0.3–0.5 Hz) and unfold within interaction bouts that recur across the day (Feldman, 2007). Feldman (2015) also demonstrated that early parent-infant synchrony predicts the development of self-regulation, symbolic competence, empathy, and attachment security across childhood and adolescence. Hyperscanning studies using fNIRS have revealed that parent-infant interaction is associated with inter-brain synchronization in prefrontal regions, with the degree of neural coupling correlating with behavioral synchrony and interaction quality (Nguyen et al., 2021). This early experience of temporally coordinated social interaction may establish the neural foundations for engaging with social rhythms at longer timescales.

Weekly rhythms represent the most ubiquitous social-frequency structure in industrialized societies. The seven-day week, though lacking astronomical basis, organizes activities like work, rest, religious observance, and social activity into a recurring pattern that modulates physiology and behavior. “Social jet lag”—the discrepancy between social schedules (especially work or school start times) and biological time preferences—accumulates across weekdays and partially resolves on weekends, creating a weekly oscillation in circadian alignment with measurable health consequences (Wittmann et al., 2006). Musical activity often follows weekly structure: ensemble rehearsals, lessons, and practice routines typically recur on weekly schedules, embedding faster rhythmic activities within this social-frequency scaffold.

Seasonal and annual rhythms organize behavior at still longer timescales. Concert seasons, academic calendars, and festival cycles structure musical activity into periods of intensive preparation, performance, and recovery that recur annually. These rhythms interact with biological seasonality: light exposure, which varies with latitude and season, modulates circadian amplitude, mood, and cognitive function through retinal projections to the SCN (Roenneberg and Merrow, 2016). Seasonal affective patterns influence motivation and energy available for sustained musical practice. At the institutional level, orchestras, schools, and conservatories operate on annual cycles that constrain and enable individual musical development.

Developmental trajectories extend social-frequency coordination across years and decades. Ericsson et al. (1993) theorized that the acquisition of musical expertise unfolds over approximately 10,000 h of deliberate practice distributed across years of training—a figure whose precision has been contested in subsequent meta-analyses (Macnamara et al., 2014), though the broader finding that structured deliberate practice drives expertise acquisition remains well-supported. This process requires sustained coordination between the developing individual and the social structures (teachers, institutions, performance opportunities) that support skill acquisition. Ensemble membership creates social rhythms at multiple timescales: weekly rehearsals, seasonal concert series, and the longer arcs of joining, developing within, and eventually leaving musical groups. A professional musician’s career trajectory, from student to emerging professional to established artist to elder statesman, represents a social-frequency rhythm unfolding across decades, shaped by individual development and the institutions that structure musical life.

The most compelling evidence for cross-timescale transfer at social frequencies comes from clinical research on Interpersonal and Social Rhythm Therapy (IPSRT) for bipolar disorder. Frank and colleagues developed IPSRT based on the “social rhythm hypothesis,” which posits that individuals with mood disorders possess vulnerability in the circadian timing system that renders them susceptible to disruption by destabilizing life events (Frank et al., 2005; Haynes et al., 2016). IPSRT focuses on stabilizing daily behavioral routines (e.g., regularizing sleep–wake times, meal times, and social activities) to strengthen circadian organization and prevent mood episodes. Multiple randomized controlled trials have demonstrated that IPSRT reduces relapse rates and improves outcomes in bipolar disorder (Frank et al., 2005; Steardo et al., 2020). This clinical evidence demonstrates that interventions targeting social-timescale rhythms produce measurable effects on circadian function, which in turn affects neural oscillatory dynamics—a clear example of downward effects across the TOE hierarchy.

The nesting relationship between social and biological frequencies completes the framework’s hierarchical architecture. Circadian rhythms are entrained by social cues (*zeitgebers*) including meal times, social contact, and work schedules, demonstrating that social-frequency structures actively shape biological timing rather than merely coinciding with it. Weekly and seasonal rhythms modulate the envelope within which circadian organization operates. At the longest timescales, developmental trajectories determine the cumulative exposure to entrainment experiences that shape an individual’s oscillatory capacity across all faster timescales. The musician who has spent decades coordinating with ensembles at multiple timescales—tracking millisecond-level onsets, breathing with phrases, rehearsing weekly, performing seasonally—has engaged in a form of temporal training that spans the full TOE hierarchy.

In ensemble terms, social frequencies encompass the career arc and the institution—the long-term relationships and structures that make sustained musical coordination possible. A professional string quartet that has played together for 20 years exhibits social-frequency coordination: their individual musical development has become intertwined, their interpretive approaches have converged through thousands of hours of shared rehearsal, and their coordination operates at timescales from milliseconds (onset synchronization) to decades (shared artistic vision). The ensemble analogy, which has served throughout this framework as an organizing metaphor, here becomes literal: musical ensemble participation is itself a social-frequency phenomenon, and the coordination it enables at faster timescales depends on the stability and continuity of these longer rhythmic structures.

## Evidence for cross-timescale transfer

The preceding sections detailed the frequency architecture of the TOE framework; the following sections examine whether the cross-frequency coupling mechanisms established earlier produce the transfer effects that the framework predicts. If the predictions hold true, training at one timescale should produce transfer effects at adjacent and potentially non-adjacent frequencies. This section reviews current evidence for such transfer, organized by the timescales involved.

### Transfer within neural frequencies

The strongest evidence for cross-frequency transfer comes from studies demonstrating that musical rhythm training at delta frequencies (approximately 1–2 Hz) transfers to phoneme discrimination at high gamma timescales (approximately 10–40 milliseconds). Besson et al. (2011), in a comprehensive review, documented that musician children with four or more years of training showed larger mismatch negativity responses and shorter reaction times to voice onset time differences—a fast temporal cue differentiating phonemes like “ba” from “pa.” Critically, non-musicians could not discriminate between small and large voice onset time deviants, suggesting that musical training enhances the precision of rapid temporal processing.

Bidelman and Alain (2015) demonstrated that musicians showed faster neural onset latencies at both subcortical and cortical levels, faster behavioral reaction times on phonemic judgment tasks, and more separable neural representations when categorizing vowel sounds. Wong et al. (2007) showed that musicians have enhanced brainstem frequency-following response tracking of linguistic pitch contours in speech. These findings indicate that musical training at beat and rhythm timescales (delta range) transfers to millisecond-level processing (high gamma range), spanning three orders of magnitude.

Additional evidence comes from studies of working memory. Yurgil et al. (2020) found that rhythmic entrainment through music listening improves working memory through enhanced theta-gamma coupling. Zanto et al. (2022) demonstrated that musical rhythm training improved short-term memory for faces, with the authors proposing that “transfer of benefit to verbal or visual short-term memory… stems from shared processes that were engaged during training” (p. 1). These findings suggest that beat tracking and temporal attention during music engage domain-general memory encoding mechanisms operating across multiple frequency bands.

### Transfer to social timescales: inter-brain synchronization

A growing body of hyperscanning research demonstrates that musical coordination produces inter-brain synchronization, providing evidence for transfer to social timescales. Müller et al. (2012) recorded EEG from pairs of guitarists playing in duets and found that phase locking and phase coherence were “enhanced at frontal and central electrodes during periods with high musical coordination demands.” Critically, they observed a frequency-dependent organization: “intrabrain synchronization operated at faster frequencies than interbrain synchronization”—within-brain connections used beta frequencies, while inter-brain connections primarily used delta and theta (p. 2667).

Hu et al. (2022) conducted fNIRS hyperscanning while participants coordinated finger-tapping with and without musical meter. They found that musical meter enhanced inter-brain synchronization at left-middle frontal cortex, and that this synchronization was “significantly correlated with participants’ performance in terms of coordination” (p. 1). Müller and Lindenberger (2023), in a study of a guitarist quartet and audience during a concert, found that inter-brain synchronization in delta band differentiated musicians from audience members and varied with musical demands.

These findings support the TOE framework’s proposal that slower oscillations coordinate activity between individuals while faster oscillations coordinate activity within individuals. Musical ensemble performance appears to engage hierarchically organized coupling mechanisms spanning from millisecond-level motor timing to social coordination at behavioral and longer timescales.

## The auditory-motor system as a privileged pathway

The auditory-motor system shows particularly strong coupling properties, making it an ideal model for studying cross-frequency coordination. Giraud and Poeppel (2012), in a foundational paper, argued for a “principled relation between the time scales present in speech and the time constants underlying neuronal cortical oscillations” (p. 511). They proposed that delta, theta, and gamma oscillations are “specifically engaged by the multi-timescale, quasi-rhythmic properties of speech and can track its dynamics,” serving to “package incoming information into units of the appropriate temporal granularity” (p. 511).

Subsequent research has revealed that auditory-motor coupling is rate-restricted. Assaneo and Poeppel (2018) demonstrated that synchronization between auditory and motor cortices during passive listening to syllable trains is maximal at approximately 4.5 syllables per second (the theta/delta boundary) and diminishes at faster or slower rates. They proposed that this reflects an “intrinsic speech-motor rhythm” that constrains auditory-motor coordination. Critically, individuals differ in their degree of auditory-motor coupling, with some showing strong synchronization (“high synchronizers”) and others showing weak coupling (“low synchronizers”). These individual differences were associated with structural differences in the arcuate fasciculus, the white matter tract connecting auditory and motor regions.

The finding of rate-restricted auditory-motor coupling has important implications for the TOE framework. It suggests that the auditory-motor system is tuned to a specific frequency range (approximately 4–5 Hz) that corresponds to natural speech rates and musical beat perception. Training that enhances synchronization at this frequency—such as musical rhythm training—might strengthen the coupling between auditory and motor systems, with potential cascading effects on both faster (phonemic) and slower (phrasal) processing.

## Musical training as a model for cross-frequency plasticity

Musical training provides an optimal model for studying cross-frequency coupling and its plasticity because it uniquely engages multiple frequency bands simultaneously. Playing a musical instrument requires precise temporal coordination at multiple timescales: millisecond-level motor timing, beat-level synchronization, phrase-level musical structure, and the social coordination of ensemble performance. Longitudinal neuroimaging studies have documented that this multi-timescale engagement produces structural and functional changes spanning subcortical to cortical levels.

Olszewska et al. (2021), in a comprehensive review, summarized evidence that musical training is associated with increased cortical thickness in the left cerebral auditory-motor network, greater gray matter volume in cerebellar regions, and higher fractional anisotropy in white matter tracts that enable auditory-motor integration, including the arcuate fasciculus, inferior longitudinal fasciculus, and corpus callosum. Critically, a study of monozygotic twins discordant for musical training (de Manzano and Ullén, 2018) demonstrated that these differences are attributable to training rather than preexisting predispositions, with the musical twin showing enhanced structural connectivity compared to their genetically identical sibling.

The most extensive longitudinal evidence comes from the AMseL (Audio and Neuroplasticity of Musical Learning) project, a 12-year study following 112 participants from primary school age through late adolescence (Schneider et al., 2023). This study combined structural MRI, magnetoencephalography, and auditory testing at five timepoints. The authors found “substantial, stable differences in the morphology of auditory cortex between musicians and nonmusicians even at the earliest ages, suggesting that musical aptitude is manifested in macroscopic neuroanatomical characteristics” (p. 6430). However, they also observed training-induced changes, with maturational plasticity leading to increased white matter myelination and systematic changes in auditory evoked responses. This interplay between predisposition and training-induced plasticity is integrated in their novel neurodevelopmental model of the human auditory system.

Neural oscillation research provides converging evidence that musical training affects multiple frequency bands. Musicians show enhanced gamma coherence between frontal and parietal cortices during spatial working memory tasks (Bhattacharya et al., 2001; Yurgil et al., 2020). Pianists demonstrate significantly greater theta power in frontal, central, and motor control areas during executive function tasks, with enhanced frontal–parietal functional connectivity in the theta band compared to string musicians and non-musicians (Wang et al., 2022). Beta oscillations are central to temporal predictions during musical beat processing, with musicians showing enhanced cortical entrainment that correlates with years of training (Doelling and Poeppel, 2015; Fujioka et al., 2015). Perhaps most strikingly, during ensemble musical performance, the brain shows frequency-dependent organization: within-brain coordination relies on faster beta frequencies, while between-brain coordination operates through slower delta and theta oscillations (Müller and Lindenberger, 2013). This hierarchical frequency organization suggests that musical ensemble experience may specifically train cross-frequency coordination mechanisms.

## Discussion

The Temporal-Oscillatory Entrainment framework synthesizes research across neural oscillations, cross-frequency coupling, auditory-motor neuroscience, chronobiology, and social rhythm research to offer a unified account of rhythmic coordination across timescales. The framework makes several contributions to understanding temporal coordination and generates testable predictions for future research.

### Theoretical contributions

The TOE framework advances existing accounts of neural oscillations in three principal ways. First, it provides an organizational taxonomy spanning from high gamma oscillations (60–200 Hz) to social frequencies (periods of weeks to years), encompassing nine frequency bands across three functional categories. While the boundaries between these bands are gradual rather than discrete, the taxonomy highlights functional distinctions obscured when oscillations are studied within narrow frequency ranges. Existing frameworks have characterized individual frequency bands in considerable detail; the TOE framework’s contribution lies in specifying how these bands coordinate through cross-frequency coupling to produce unified perception, cognition, and action.

Where Dynamic Attending Theory proposed that attention entrains to external rhythms, TOE specifies the mechanism (cross-frequency coupling), extends the timescale range (neural through social frequencies), and identifies the training pathway (auditory-motor system). The framework thus integrates DAT’s foundational insights with subsequent neuroscience to generate predictions about cross-timescale transfer that DAT’s original formulation did not address.

Second, the framework proposes that musical ensemble coordination *instantiates* the same organizational logic as neural cross-frequency coupling. This claim warrants careful articulation; the argument is not that ensemble performance is a useful metaphor for understanding neural dynamics—though that is also true. The hierarchical nesting of timescales, the phase-based coordination across temporal distances, and the bidirectional influence between fast and slow processes characterize both the neural and musical domains because both face the same computational problem: coordinating activity across timescales that differ by orders of magnitude. The conductor’s downbeat and the delta oscillation serve homologous functions because both solve the problem of providing a temporal scaffold within which faster processes can unfold with local autonomy while remaining globally coordinated.

This structural correspondence has implications beyond analogy. If musical ensemble performance engages the same coordination mechanisms that organize neural activity, then ensemble experience constitutes direct training of cross-frequency coupling. A musician who spends decades coordinating millisecond-level onsets with phrase-level structure with weekly rehearsal schedules is exercising temporal coordination capacities across the full range of the TOE hierarchy. The framework thus provides a principled account of why musical training produces transfer effects to specific non-musical domains: music specifically trains the coupling mechanisms that bind neural processes across timescales. The transfer is domain-specific by design: it extends to tasks that draw on the same coupling architecture that musical training engages, and the framework makes no prediction about domains that do not.

A clarification about the framework’s scope is warranted here. TOE does not propose temporal coordination as the primary organizing principle of cognition broadly, nor does it claim to displace established accounts of semantic processing, executive function, social cognition, or other cognitive domains. The framework’s claim is that temporal coordination is a foundational capacity in the sense that many cognitive functions depend on it without being reducible to it. Semantic processing requires that phonological units be parsed at appropriate timescales; executive function requires the sequencing of actions across time; social cognition depends on the temporal coordination of interaction. TOE addresses the temporal substrate these functions share, not the content-specific mechanisms that distinguish them. The framework is therefore best understood as operating alongside, rather than in competition with, domain-specific cognitive accounts—specifying the temporal architecture within which those domains operate rather than supplanting their explanatory contributions.

Third, the TOE framework integrates findings from respiratory-brain coupling research to demonstrate that the proposed bridges between neural and biological timescales are mechanistically grounded. Respiration, operating at approximately 0.2–0.5 Hz, modulates neural oscillations through phase-amplitude relationships structurally similar to those observed within neural frequencies. This integration extends the principle of hierarchical nesting beyond the brain proper, suggesting that the temporal architecture described by the TOE framework encompasses bodily rhythms traditionally studied separately from neural oscillations. Tort et al.'s (2018) finding that respiratory phase couples to neural oscillations across distributed brain regions is evidence that cross-frequency coupling operates across the neural-biological boundary. It is worth noting that PAC in its strict technical sense (the phase of a slow oscillation modulating the amplitude of a faster oscillation, as documented in hippocampal and cortical recordings) is the best-characterized mechanism at neural frequencies. At biological and social timescales, the TOE framework invokes the same organizational logic (hierarchical nesting, phase-based coordination, bidirectional influence across levels) rather than claiming that PAC itself operates identically across all tiers. The correspondence here is architectural: the framework proposes that the coordinative principles instantiated by PAC within neural circuits extend outward to encompass bodily and social rhythms, whether or not the precise molecular and cellular mechanisms of PAC are the mediating substrate at those slower scales.

### The auditory-motor system and the privileged status of musical training

The TOE framework identifies the auditory-motor system as a privileged pathway for cross-frequency coupling. This privilege is anatomical: the frequency-following response demonstrates subcortical CFC in the auditory brainstem, and this response shows training-dependent plasticity. Other forms of rhythmic experience (e.g., dance, athletic coordination, breathing practices) engage temporal coordination through cortical routes, but they do not access subcortical CFC mechanisms with the directness that auditory-motor training provides. This anatomical specificity helps explain why musical training produces transfer effects to domains (phoneme discrimination, speech-in-noise perception) that depend on brainstem processing precision.

Musical training engages this privileged pathway with specificity and intensity. Playing an instrument requires precise auditory-motor coordination at the system’s preferred frequency range while simultaneously demanding integration with faster (articulatory, timbral) and slower (phrasal, formal) timescales. Ensemble performance adds the social dimension: inter-brain synchronization at delta and theta frequencies, documented in hyperscanning studies, demonstrates that musical coordination extends the auditory-motor coupling from an intrapersonal to an interpersonal phenomenon. The musician in an ensemble is training both their own cross-frequency coupling and their capacity to couple with other nervous systems operating at the same timescales.

This analysis suggests studies examining only individual musicianship do not fully test the framework’s predictions about social-frequency coordination. Solo practice engages auditory-motor coupling and cross-frequency coordination within the individual; ensemble experience extends this training to the social-frequency domain. The TOE framework predicts that ensemble musicians should show enhanced inter-brain synchronization capacity compared to soloists matched for years of training—a testable hypothesis with implications for understanding the distinct contributions of individual and collaborative musical experience.

### Clinical implications

The finding that disrupted cross-frequency coupling characterizes multiple psychiatric and neurological disorders (Yakubov et al., 2022) suggests therapeutic applications for the TOE framework. If CFC disruption contributes to symptomatology in autism spectrum disorder, schizophrenia, Parkinson’s disease, and mood disorders, then interventions that strengthen cross-frequency coordination might have clinical value. The framework provides a principled basis for understanding why rhythm-based interventions have shown promise across these conditions through targeted training of the coupling mechanisms that appear disrupted.

The social rhythm therapy literature provides the strongest existing clinical evidence for cross-timescale transfer. Frank and colleagues’ demonstration that stabilizing daily behavioral routines affects circadian function and mood regulation in bipolar disorder (Frank et al., 2005) represents intervention at social-frequency timescales producing effects at biological and, ultimately, neural timescales. The TOE framework situates this clinical finding within a broader theoretical architecture, suggesting that similar downward-propagating effects might be achievable through interventions at other points in the hierarchy.

Musical training emerges as a candidate intervention with promising properties, though its clinical rationale requires careful delineation. Unlike social rhythm therapy, which targets behavioral regularization, musical training engages active cross-frequency coordination across multiple timescales simultaneously. Unlike pharmacological interventions, which typically target specific neurotransmitter systems, musical training may strengthen the coupling mechanisms that coordinate activity across neural populations. The clinical value of this pathway is specific rather than general: populations whose symptomatology involves disrupted temporal coordination (e.g., timing deficits in autism spectrum disorder, rhythm perception impairments in Parkinson’s disease) are likely more directly targeted by the TOE framework’s predictions than conditions where CFC disruption is more diffuse or less temporally specific. Music is not a broad-spectrum cognitive enhancer; it is a temporally structured activity that directly trains the coupling mechanisms the framework identifies as disrupted in particular conditions. This specificity is a feature rather than a limitation: it generates testable predictions about which populations and symptom profiles should be most responsive, and it constrains the claims that can appropriately be made on behalf of rhythm-based intervention.

### Testable predictions

The TOE framework generates several testable predictions that distinguish it from general claims about the benefits of rhythmic experience. First, training at any frequency band should show bidirectional transfer effects, thereby enhancing both faster and slower processing. Existing evidence supports transfer from delta-frequency beat training to gamma-timescale phoneme discrimination, but systematic studies of transfer to slower timescales are lacking. The framework predicts, for example, that rhythm training should affect respiratory-neural coupling (behavioral frequencies) and that respiratory training should affect neural oscillatory dynamics. These predictions are falsifiable and would, if disconfirmed, require modification of the framework’s core claims about bidirectional coupling.

Second, the strength of cross-frequency coupling should predict the magnitude of transfer effects. Individuals with stronger theta-gamma coupling at baseline should show greater transfer from rhythm training to working memory performance. This prediction follows directly from the framework’s proposal that CFC provides the mechanism of transfer; if coupling strength does not predict transfer magnitude, the mechanistic account requires closer examination.

Third, ensemble musical experience should produce effects on inter-brain synchronization capacity that solo musical experience does not. The framework’s claim that social-frequency coordination extends the logic of neural CFC to the interpersonal domain predicts that training in coordinating with others should enhance the capacity for such coordination—an effect distinct from the auditory-motor coupling benefits of individual practice.

Fourth, individual differences in entrainment capacity that reflect baseline precision, coupling strength, and training-induced plasticity, should predict learning outcomes across domains requiring temporal coordination. The finding that individuals differ in auditory-motor coupling strength (Assaneo and Poeppel, 2018) suggests that some individuals may benefit more from rhythmic training than others. Characterizing the sources of this variation (e.g., genetic, developmental, experiential) and its consequences for training response represents a priority for future research.

Fifth, circadian effects of rhythmic training should be detectable with appropriate chronobiological measures. Preliminary evidence from music therapy studies on melatonin (Kumar et al., 1999) suggests that rhythmic interventions can affect circadian markers, but controlled studies with active musical training in healthy populations are needed. The framework predicts that regular musical practice, particularly when scheduled at consistent times, should strengthen circadian amplitude—a prediction testable through actigraphy, hormonal assays, and chronotype assessment.

## Limitations

The current framework has several limitations. First, the evidence for transfer effects at biological and social timescales is currently weaker than the evidence at neural timescales. Extant literature examines musician advantages in phoneme discrimination, working memory, and related cognitive functions provides robust support for transfer within neural frequencies. The evidence that musical training affects ultradian, circadian, or social-frequency organization is more limited, resting primarily on the IPSRT literature (which involves behavioral regularization rather than musical training) and preliminary studies of music therapy effects on circadian markers. Longitudinal studies examining whether musical training affects biological and social rhythms represent a priority for establishing the framework’s claims across the full hierarchy.

Second, the dose–response relationship between training intensity and transfer effects remains unexplored. While cross-sectional studies document correlations between years of musical training and neural measures, these studies cannot establish causation and do not characterize the minimum effective dose. A discordant twin study (de Manzano and Ullén, 2018) provides evidence that training rather than preexisting predisposition accounts for at least some musician-nonmusician differences, but the training histories of discordant twins are difficult to control. Randomized controlled trials with varying training intensities would strengthen causal inference.

Third, the framework’s emphasis on cross-frequency coupling as the mechanism of transfer, while grounded in substantial neuroscience literature, remains partially speculative at the level of specific training effects. Demonstrating that rhythm training affects CFC measures and that changes in CFC mediate transfer effects would provide stronger evidence than the current reliance on plausibility arguments from the CFC literature.

Fourth, the boundaries between frequency bands in the TOE taxonomy are gradual rather than discrete, and the nine-band organization, while useful for exposition, may impose categorical structure on continuous phenomena. Future work should examine whether the proposed boundaries correspond to genuine discontinuities in coupling mechanisms or represent convenient but arbitrary divisions of a continuous spectrum.

Fifth, any advantages that musical training provides are not necessarily broad, real-world cognitive advantages. The TOE framework draws on evidence that musical training produces specific transfer effects to tasks requiring temporal coordination precision: phoneme discrimination, speech-in-noise perception, working memory for sequenced information, and sensorimotor synchronization (Besson et al., 2011; Bidelman and Alain, 2015; Wong et al., 2007; Yurgil et al., 2020). This evidence is appropriate for the framework’s mechanistic claim—musical training strengthens cross-frequency coupling through the auditory-motor pathway, with downstream benefits in domains that functionally depend on those mechanisms. It is not evidence for (and the framework does not predict) broad real-world cognitive advantages in domains unrelated to temporal coordination. The absence of observational data showing that musicians are generally smarter, more academically successful, or professionally advantaged is not a limitation of the TOE framework. These are different claims addressing different questions, and we should not apply the evidentiary standard for one to the other. The genuine limitation here is that even the domain-specific transfer evidence derives predominantly from laboratory tasks, and controlled longitudinal studies examining real-world outcomes in domains requiring temporal precision (e.g., auditory processing in degraded environments or tasks requiring fine-grained sequential coordination) remain limited.

## Future directions

Addressing these limitations requires research programs spanning multiple methodologies and timescales. Longitudinal intervention studies should assess transfer effects across the full TOE hierarchy, employing measures appropriate to each timescale: EEG and MEG for neural frequencies, respiratory and cardiac monitoring for behavioral frequencies, actigraphy and hormonal assays for circadian rhythms, and ecological momentary assessment for social-frequency patterns. Such studies would test whether musical training produces the cross-timescale transfer effects the framework predicts.

Individual differences research should characterize variation in entrainment capacity and its sources. Combining genetic approaches (twin studies, candidate gene analyses), developmental approaches (longitudinal tracking from infancy), and experiential approaches (detailed training histories) would illuminate why some individuals show stronger CFC and greater training response than others.

Clinical trials should examine whether rhythm-based interventions affect CFC measures in populations with documented coupling disruptions, and whether changes in CFC mediate symptom improvement. Such studies would test the framework’s therapeutic implications while advancing understanding of oscillatory mechanisms in psychopathology.

Comparative research on ensemble versus solo musical training would test the framework’s prediction that interpersonal coordination produces distinct effects on inter-brain synchronization capacity. Hyperscanning studies comparing musicians with matched years of solo versus ensemble experience would provide relevant evidence.

Finally, the development of standardized measures of entrainment capacity would facilitate research across these domains. Currently, many studies use heterogeneous paradigms that make cross-study comparison difficult. Consensus measures of beat perception, sensorimotor synchronization, cross-frequency coupling strength, and inter-brain synchronization capacity would accelerate progress.

## Conclusion

The Temporal-Oscillatory Entrainment framework proposes that temporal coordination is a fundamental organizing principle of neural function, operating through cross-frequency coupling mechanisms that span from millisecond-scale spike timing to the social rhythms structuring daily life. The framework’s central claim is that these timescales are both concurrent and hierarchically integrated: slow oscillations provide temporal scaffolds within which faster processes unfold, and training at any point in the hierarchy can propagate effects both upward and downward through the nested structure.

Musical ensemble performance emerges as a distinctive form of temporal training. The musician coordinating with an ensemble exercises cross-frequency coupling across the full TOE hierarchy: millisecond-level onset precision, beat-level synchronization, phrase-level structure, rehearsal-level learning, and the longer arcs of musical development unfolding across years. The auditory-motor system’s privileged status as a coupling pathway—its rate-restricted tuning to the frequency range of musical beat processing—positions musical training as optimally suited to strengthen the coupling mechanisms that the framework identifies as foundational to temporal coordination.

This analysis reframes longstanding questions about music and cognition. The question is not whether music has generic ‘transfer effects’ to unrelated domains. Instead, the question is whether temporal coordination is a foundational capacity for the cognitive functions that depend on it, whether cross-frequency coupling provides its mechanism, and whether musical training strengthens that mechanism in ways that transfer to other temporally-demanding tasks.

The framework has implications for education, clinical intervention, and basic science. For education, it provides a principled rationale for musical training if temporal coordination is itself a foundational capacity warranting development. For clinical intervention, it identifies rhythm-based approaches as targeting specific mechanisms (e.g., cross-frequency coupling) rather than operating through nonspecific enrichment. For scientific research, it generates testable predictions about cross-timescale transfer, individual differences in entrainment capacity, and the contributions of solo and ensemble musical experience.

The musical ensemble comparison with which this paper opened is more than analogy. The nervous system and musical ensemble coordinate activity across timescales through hierarchical nesting and phase-based coupling. Both face the same challenge—integrating processes operating at vastly different speeds into coherent, adaptive wholes—and both solve it through the same organizational logic. Understanding this correspondence illuminates both how the brain achieves temporal coordination and why engaging in musical coordination might strengthen the capacity to do so. The conductor’s downbeat and the delta oscillation are solutions to this same problem, implemented in different substrates but governed by the same principles. To train coordination at one level is, in a meaningful sense, to train it at others.

## Data Availability

The original contributions presented in the study are included in the article/supplementary material, further inquiries can be directed to the corresponding author.
